# The LeucoPatch® system in the management of hard-to-heal diabetic foot ulcers: study protocol for a randomised controlled trial

**DOI:** 10.1186/s13063-017-2216-9

**Published:** 2017-10-10

**Authors:** Frances Game, William Jeffcoate, Lise Tarnow, Florence Day, Deborah Fitzsimmons, Judith Jacobsen

**Affiliations:** 1 Derby Teaching Hospitals NHS Foundation Trust, Derby, UK; 2 Foot Ulcer Trials Unit, Nottingham University Hospitals NHS Trust, Nottingham, UK; 3 Nordsjællands Hospital, Hillerød, Denmark; 4 Nottingham Clinical Trials Unit, University of Nottingham, Nottingham, UK; 5 Swansea Centre for Health Economics, Swansea University, Swansea, UK; 6 Magnus Löndahl, Skåne University Hospital, Lund, Sweden

**Keywords:** Diabetic foot ulcer, Ulcer healing, Diabetes complications, Amputation, Platelets

## Abstract

**Background:**

Diabetic foot ulcers are a common and severe complication of diabetes mellitus. Standard treatment includes debridement, offloading, management of infection and revascularisation where appropriate, although healing times may be long. The LeucoPatch® device is used to generate an autologous platelet-rich fibrin and leucocyte wound dressing produced from the patient’s own venous blood by centrifugation, but without the addition of any reagents. The final product comprises a thin, circular patch composed predominantly of fibrin together with living platelets and leucocytes. Promising results have been obtained in non-controlled studies this system, but this now needs to be tested in a randomised controlled trial (RCT). If confirmed, the LeucoPatch® may become an important new tool in the armamentarium in the management of diabetic foot ulcers which are hard-to-heal.

**Methods:**

People with diabetes and hard-to-heal ulcers of the foot will receive either pre-specified good standard care or good standard care supplemented by the application of the LeucoPatch® device.

The primary outcome will be the percentage of ulcers healed within 20 weeks. Healing will be defined as complete epithelialisation without discharge that is maintained for 4 weeks and is confirmed by an observer blind to randomisation group.

**Discussion:**

Ulcers of the foot are a major source of morbidity to patients with diabetes and costs to health care economies. The study population is designed to be as inclusive as possible with the aim of maximising the external validity of any findings. The primary outcome measure is healing within 20 weeks of randomisation and the trial also includes a number of secondary outcome measures. Among these are rate of change in ulcer area as a predictor of the likelihood of eventual healing, minor and major amputation of the target limb, the incidence of infection and quality of life.

**Trial registration:**

International Standard Randomised Controlled Trial, ISRCTN27665670. Registered on 5 July 2013.

## Background

### Diabetic foot ulcers

Diabetic foot ulcers (DFU), a common and severe complication of diabetes mellitus, are currently the most common chronic type of wound in Western industrialised countries [1]. Despite improved outcomes following modern standard treatment, DFUs are still the predominant reason for non-traumatic leg amputation in most Western countries, amputation rates in patients with diabetes being described as much as 15-fold higher than those in non-diabetic populations [2].

Diabetic foot ulceration is the consequence of neuropathy and micro- and macrovascular disease [3]. The syndrome is frequently worsened by infection [4].

As all aspects of this multifactorial aetiology must be taken into account, modern standard treatment includes regular debridement, off-loading, treatment of infection, revascularisation when appropriate, optimising metabolic control and the treatment of any concomitant diseases, as well as education about foot care and the provision of appropriate footwear [5, 6].

If these treatment strategies are properly applied the majority of chronic non-healing ulcers will heal, although healing times may be prolonged [1].

The presence of a DFU is also associated with significant reduction in health-related quality of life (HRQoL); ulcer healing is associated with improved HRQoL [7, 8].

### Growth factors and healing

Growth factors are involved throughout the healing process [9–11]. At the cellular level growth factors mediate macrophage migration, neovascularisation, collagen synthesis, fibroblast proliferation as well as epithelialisation [12–15].

Animal, as well as in-vitro studies, have shown positive effects of platelet-derived growth factors on ulcer healing [16, 17]. Platelets contain a range of potent growth factors. These consist, among others, of platelet-derived growth factor (PDGF), transforming growth factor-β (TGF-β), epidermal growth factor (EGF) and vascular endothelial growth factor (VEGF), which together exhibit different chemotactic, mitogenic and proliferative properties.

The first clinical study evaluating the effect of platelet-derived growth factors on ulcer healing was performed by Knighton et al. in 1986 [18]. In this small, prospective, randomised, blinded, placebo-controlled, cross-over study the effect of a platelet-derived, wound-healing formula (PDWHF) was evaluated. The frequency of complete epithelialisation of ulcers was significantly higher in PDWHF-treated patients when compared to controls (87% versus 15%), and, after cross-over, complete epithelialisation was achieved in the control group after 7.1 weeks [18]. In a case-series of 24 patients, Crovetti et al. achieved sustained ulcer healing in eight patients with chronic cutaneous ulcers, and ulcer area reduction in another seven cases, after topical treatment with a platelet gel [19].

In a retrospective cohort study, Margolis et al. estimated the effectiveness of platelet releasate in the treatment of diabetic neuropathic foot ulcers using the Curative Health Services database including 120,000 patients with chronic wounds. The analysis suggested that treatment with platelet releasate was more effective than standard therapy, with the most pronounced beneficial effect being seen in more serious ulcers [20]. However, Stacey et al. found no effect on the healing of chronic venous leg ulcers using lysed platelets in a randomised, double-blind, placebo-controlled study [21]. Neither could Senet et al. show any beneficial effect on healing of chronic venous leg ulcers [22].

Since the study by Knighton et al., many methods for producing platelet releasate, platelet gel and fibrin-platelet suspensions have been developed. They are all quite complex and all require the addition of different reagents, and thus cannot by definition be characterised as purely autologous products. The concentration and activation of platelets in these products varies and, despite promising early results, the need for confirmation in large, good-quality randomised controlled trials (RCTs) remains [6].

### LeucoPatch®

**Fig. 1:**
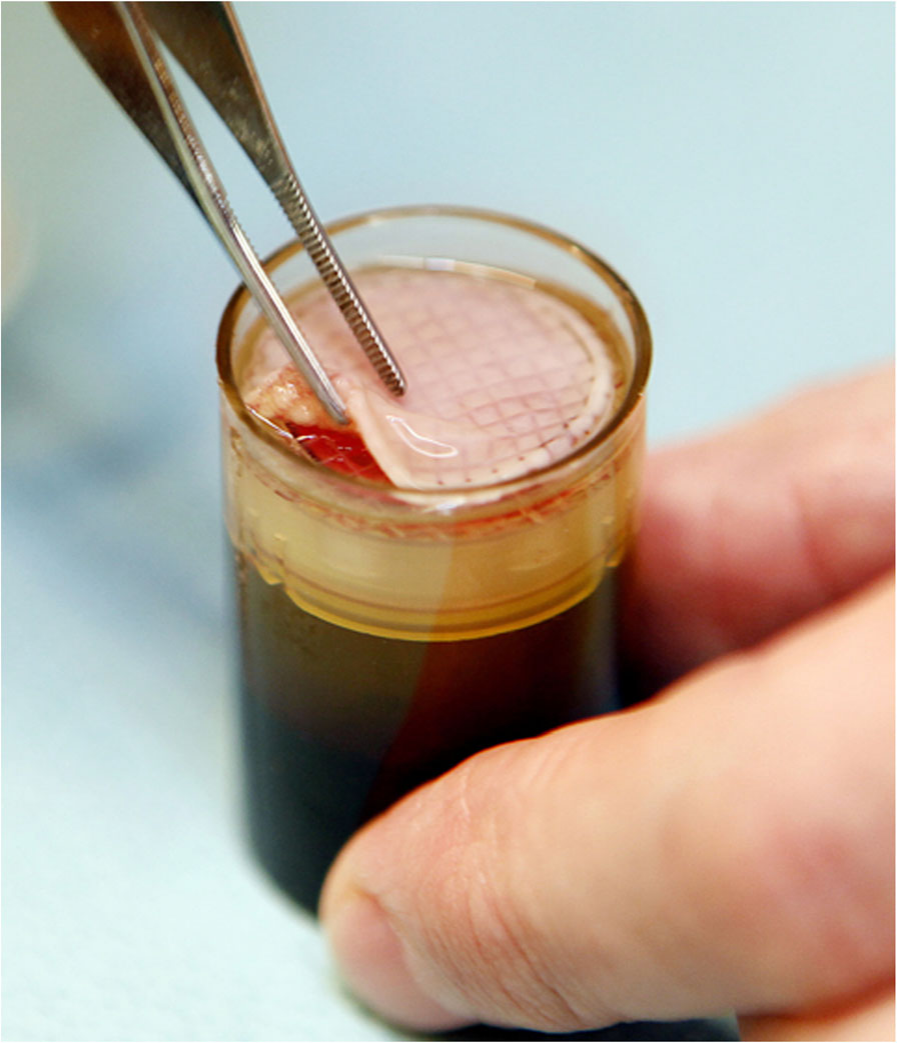
A LeucoPatch® post centrifugation

The LeucoPatch® device is a CE-marked, single-use medical device used to generate an autologous platelet- and leucocyte-rich fibrin wound dressing named the LeucoPatch®. A LeucoPatch® is produced from the patient’s own venous blood by centrifugation, but without the addition of any reagents. The final product comprises a thin, circular patch composed predominantly of fibrin together with living platelets and leucocytes (Fig. 1). The number of LeucoPatches® applied can be adjusted to fit the individual wound. The yield of platelets is close to 100% and varies minimally from patient to patient. The content and release of growth factors of this product is equal to, or higher, than other reported preparations. The product differs from other autologous platelet products by containing a high concentration of fibrin as well as both platelets and leucocytes.

In an initial pilot study, promising beneficial effects on ulcer healing after the application of the LeucoPatch® were seen [23]. Following this, in an open, clinical multicentre study including 41 patients with hard-to-heal DFUs (median ulcer duration of 40 weeks) an intention-to-treat (ITT) analysis showed that 12 (32%) healed within 12 weeks of once-weekly treatment and 22 (54%) healed within 20 weeks. No safety issues were raised in this trial [24]. This apparent beneficial effect now needs to be tested in a RCT. If confirmed, the LeucoPatch® may become an important new tool in the armamentarium for the management of DFUs which are hard-to-heal.

### Aims

**Fig. 2:**
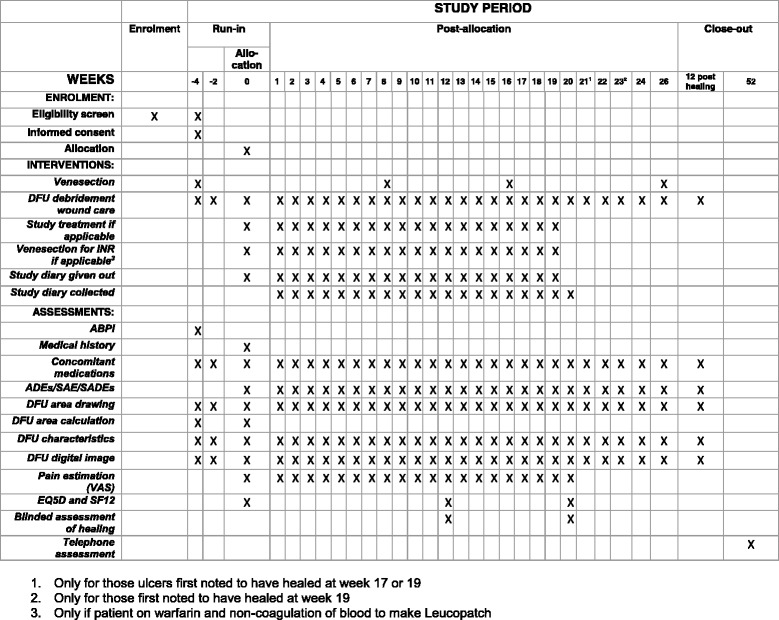
Standard Protocol Items: Recommendations for Interventional Trials (SPIRIT) Checklist

This study will demonstrate whether the application of the LeucoPatch®, when used in addition to usual care in a multidisciplinary, diabetes foot clinic setting, is superior to usual care alone with regard to complete epithelialisation of hard-to-heal DFUs which are not infected at the time of randomisation. The Standard Protocol Items: Recommendations for Interventional Trials (SPIRIT) schedule is given in Fig. 2.

## Methods

### Design

This is a multicentre, multinational, observer-blind, randomised controlled trial. Patients will be randomised 1:1 to the intervention or the usual-care arm.

### Study setting and participants

Participants will be recruited from specialist diabetes foot care clinics at sites in the UK, Denmark and Sweden.

Eligible participants will be people aged 18 years and over who have diabetes complicated by one or more foot ulcers. In addition, the following inclusion and exclusion criteria will apply:

### Inclusion criteria

- Eligible ulcers will be below the level of the malleoli, excluding ulcers confined to the interdigital cleft
- Eligible ulcers will be hard-to-heal, meaning that the cross-sectional area will decrease by less than 50% during a 4-week run-in period
- The cross-sectional area of the index ulcer will be ≥ 50 and ≤ 1000 mm^2^ at the end of the 4-week run-in period
- At randomisation, the index ulcer will be clinically non-infected according to the Infectious Diseases Society of America (IDSA) criteria
- Either the Ankle-brachial Pressure Index (ABPI) in the affected limb will be between 0.50 and 1.40 or the dorsalis pedis pulse and/or the tibialis posterior pulse will be palpable
- Glycosylated haemoglobin (HbA_1c_) level ≤ 108 mmol/mol at screening
- Participants will have the capacity to understand study procedures, and will be able to provide written informed consent

### Exclusion criteria

- Increase in cross-sectional area of the index ulcer by ≥ 25% during the 4-week run-in period, or is either smaller than 50 mm^2^ or larger than 1000 mm^2^ at the end of that time
- Clinical signs of infection of the index ulcer or reason to suspect that infection is present at randomisation
- Revascularisation procedure in the affected limb planned, or undertaken within the 4 weeks prior to screening
- Treatment of foot ulcers with growth factors, stem cells or equivalent preparation within the 8 weeks prior to screening
- The need for continued use of negative-pressure wound therapy
- Haemoglobin concentration < 105 g/L or 6.5 mmol/L at screening
- Presence of sickle-cell anaemia, haemophilia, thrombocytopenia (<100 × 10^9^/L) or other clinically significant blood dyscrasia
- Known potential infectivity of blood products, including known HIV and hepatitis
- Dialysis or an estimated glomerular filtration rate (GFR) (based on cystatin C or serum creatinine) < 20 ml/min/1.73 m^2^
- Current treatment with cytotoxic drugs or with systemically administered glucocorticoids or other immunosuppressants. Likely inability to comply with the need for weekly visits because of planned activity
- Participation in another interventional clinical foot ulcer-healing trial within the 4 weeks prior to screening
- Prior randomisation in this trial
- Judgement by the investigator that the patient does not have the capacity to understand the study procedures or to provide written informed consent

### Sample size

Previous non-controlled LeucoPatch® outcome data suggested a healing rate (intention-to-treat) of 54% during a 20-week follow-up period in a study population similar to this study population. Outcome data from a matched control group as well as from placebo/control groups in other diabetic foot studies with inclusion and exclusion criteria similar to these criteria indicate healing rates between 27 and 32% at 20 weeks’ follow-up, although some authors have reported healing rates below 10%.

A sample size for comparing two proportions with Fleiss continuity correction, based on alpha = 0.05 and beta = 80% and with an outcome rate in the control group of 30% and an improvement of 18 percentage points (i.e. to 48%) in the treatment group gives a sample size of 250 evaluable patients. To allow for 30% dropout a sample size of 350 randomised participants is sought.

As there is uncertainty on both healing rates and dropout rates, the Data Monitoring Committee (DMC) will perform an interim based re-estimation of sample size when 140 randomised patients (70 patients in each group) have completed 20 weeks of follow-up. The sample size re-estimation will be done by comparing 20 weeks’ healing proportions from two groups with Fleiss continuity correction, based on two-sided alpha = 0.04 and beta = 80%, on both the intention-to-treat (ITT) and per-protocol (PP) populations.

### Interventions

All participants will receive pre-specified good standard care of their diabetic foot ulcers in a multidisciplinary diabetes foot clinic setting, following international guidelines [5] as described in Table 1.

**Table 1:** Components of good standard wound care

• Formal assessment of ulcer and surrounding skin	
• Provision of any necessary off-loading	
• Debridement (i) sharp, (ii) other as appropriate (but excluding the use of larvae)	
• Appropriate dressing products	
• Appropriate antibiotic therapy	
• Nutrition and self care	
• Optimal glycaemic control	
• Revascularisation if deemed clinically necessary	
• Continued close observation	

Participants randomised to the intervention group will, in addition, receive a weekly application of the LeucoPatch®. The LeucoPatch® is prepared by centrifuging one (for ulcers ≤ 5 cm^2^) or two (for ulcers > 5 cm but ≤ 10 cm^2^) 18-mL aliquots of the participant’s venous blood according to the instructions for use. The centrifugation yields a tough layer of fibrin, with viable leucocytes and platelets, and this is applied directly to the wound surface using sterile forceps. The wound is then covered with an inert primary dressing, a secondary protective dressing and the ulcerated area protected with appropriate off-loading.

### Study visit schedule (Fig. 2)

After giving written informed consent participants will be seen every 2 weeks during a 4-week run-in period to confirm eligibility. Those fulfilling inclusion and exclusion criteria at the end of the run-in period will be randomised and receive study treatment once weekly for 20 weeks or until healing, whichever occurs first. When a blinded observer has confirmed healing of the target ulcer, the patient will be scheduled for two healing confirmatory visits within the next 4 weeks. If target ulcer healing is confirmed by a blinded assessor at the 4-week confirmatory visit, the participant will enter the follow-up phase of the study, completing follow-up visits at 20 and 26 weeks post randomisation. If the target ulcer remains unhealed after 20 weeks of treatment, the participant will complete follow-up visits at 20 and 26 weeks post randomisation.

An additional study visit 12 weeks after initial healing and a telephone follow-up at 52 weeks after randomisation will be made to collect ulcer status (durability of wound healing) and safety information.

### Outcomes

#### Primary outcome

Comparison of the number (%) of hard-to-heal ulcers that heal within 20 weeks following pre-specified good standard care with the numbers healed following standard care plus the topical application of LeucoPatch® in a multidisciplinary diabetes foot clinic setting.

Healing will be assessed following any necessary debridement and will be defined as complete epithelialisation, which is maintained for 4 weeks. Healing will be confirmed both at the start and the end of the 4-week period by an observer who is blind to randomisation group. The date of healing will be that at which it was first noted by the clinical researcher and confirmed by an observer, blind to the intervention group.

#### Secondary effectiveness outcomes

Ulcer-related outcomes:

- Time (days) to healing by 20 weeks
- The incidence of healing within 12 and 26 weeks
- Change in ulcer area at 4, 12, 16, 20 and 26 weeks (as compared to week 0), as assessed by acetate tracing and digital images, measured by a blinded assessor
- Change in ulcer healing rate between the run-in period and the first 4 weeks in the treatment period
- The incidence of secondary infection
- Number of days of systemic antibiotic therapy administered for foot ulcer infection during the 20 weeks from randomisation
- Durability of wound healing 12 weeks after complete wound healing

Patient-related outcomes:

- The incidence of major (above-ankle) amputation affecting the target limb by 12, 20 and 26 weeks
- The incidence of major amputation affecting the contralateral limb by 26 weeks
- The incidence of minor (below-ankle) amputation affecting the target limb by 12, 20 and 26 weeks
- The incidence of minor amputation affecting the contralateral limb by 26 weeks
- Quality-of-life measured as using the Short Form 12 questionnaire (SF-12) and EuroQol-5D questionnaire (EQ-5D) at baseline, 12 and 20 weeks
- Pain as assessed by Visual Analogue Scale (VAS)
- Incidence of new anaemia

Patients enrolled in the study will be asked to keep a patient diary to inform a health economic record by logging information on:

- Consultations, mode and place of consultations with health care professionals not scheduled for the purpose of the study
- Usage of home help or home nursing
- Time spent travelling for consultations scheduled and not scheduled for the purpose of the study
- Direct cost of travelling for consultations scheduled and not scheduled for the purpose of the study
- Days off work due to the foot ulcers
- Assistance from non-health care professionals due to foot ulcers

Centres enrolled in the study will be asked to log:

- Average time per staff category/grade spent per patient administrating standard care and standard care with the LeucoPatch® used in addition
- Average salary and working hours for staff categories involved in the study
- Current medications, dressings and devices used for the treatment of all patients included in the study as detailed under the study visit schedule

### Health economic evaluation

Specific objectives are to establish the costs of the LeucoPatch® in addition to usual care and assess the cost-effectiveness of the LeucoPatch® in addition to usual care versus usual care alone. The perspective of the health care system (e.g. UK NHS) and personal social services, with consideration of a broader societal perspective which considers the impact on patients and their families will be taken. As the study involves three countries, appropriate consideration, following good practice guidelines [25], will be taken to ensure that resources and costs are appropriately calculated given the potential for differences in resource use between countries. Costs associated with the intervention will be determined by calculating the cost of staff time, materials and other resources involved in providing the intervention. These will be compared with changes in the number of visits to general practitioners, hospital, prescribed medication, and social services’ contacts in the intervention and control groups during the investigation. In addition, should data allow, the costs incurred by patients and their families (e.g. travel, time off work) will be calculated. The costs will be compared with the outcomes generated and a series of incremental cost-effectiveness ratios computed, including a cost/quality-adjusted life year analysis, based on changes in EQ-5D. A series of one-way sensitivity analyses will be undertaken to determine the extent to which baseline findings change in light of parameter variation and a probabilistic sensitivity analysis undertaken to determine the extent to which the intervention can be regarded as representing value for money. Should evidence be found from the within-trial analysis on the short-term effectiveness of LeucoPatch® in addition to usual care compared to usual care alone, a decision analytical model will be developed in order to assess cost-effectiveness over a longer time horizon.

Patients enrolled in the study will be asked to keep a patient diary to inform a health economic record by logging information on:

- Consultations, mode and place of consultations with health care professionals not scheduled for the purpose of the study
- Usage of home help or home nursing
- Time spent travelling for consultations scheduled and not scheduled for the purpose of the study
- Direct cost of travelling for consultations scheduled and not scheduled for the purpose of the study
- Days off work due to the foot ulcers
- Assistance from non-health care professionals due to foot ulcers

Centres enrolled in the study will be asked to log:

- Average time per staff category/grade spent per patient administrating standard care and standard care with the LeucoPatch® used in addition
- Average salary and working hours for staff categories involved in the study
- Current medications, dressings and devices used for the treatment of all patients included in the study as detailed under the study visit schedule

### Safety and tolerability measures

Safety will be assessed in relation to adverse events (AEs), serious adverse events (SAEs), adverse device effects (ADEs) and serious adverse device effects (SADEs).

AEs will be assessed for relatedness to the device, including device failures, errors or misuse of the device. For the purposes of assessing relatedness, the ‘device’ is defined as the three components of the CE-marked LeucoPatch® system, that is:

- The LeucoPatch® device
- The LeucoPatch® needle holder
- Centrifuge insert

No adverse events are expected as a result of the use of the device.

### Analyses

All analyses will be completed on both the ITT and the PP populations. The primary analysis for outcome will be a logistic regression for the proportions of patients healed within 20 weeks taking into account ulcer area, ulcer depth and ABPI.

All secondary variables will be presented using appropriate descriptive statistics and analysed on the basis of the level of measures and the distribution of scores (where appropriate). Analyses will include survival analysis for time to healing and general linear models for difference in change of pain score; with EQ-5D and SF-12 data presented in line with the conventions for these tools.

Cost-effectiveness and cost-utility analysis will be performed based on the cost-related endpoints collected in the study.

### Randomisation and blinding

Internet-based treatment assignment will be determined by a computer-generated random code using random permuted blocks of randomly varying size, created by the Nottingham Clinical Trials Unit in accordance with their Standard Operating Procedures. Trial participants will be allocated with equal probability to each treatment arm with stratification by centre, and by ulcer area (≤ 100 mm^2^ versus > 100 mm^2^).

The use of the LeucoPatch® in the intervention group will be apparent to both the participant and health professionals involved in the care of their foot ulcer. The primary outcome will, however, be confirmed by observers blind to randomisation group and all other outcomes will be analysed by researchers blind to randomisation group.

## Discussion

Ulcers of the foot are a major source of morbidity to patients with diabetes and costs to health care economies. Whilst clinicians have a plethora of topical and systemic treatments to choose from, few have been subjected to rigorous evaluation in a blinded randomised trial [6].

Early studies with growth-factor-based [18] methods for producing platelet releasate, platelet gel or fibrin-platelet suspensions have been complex and/or required large blood volumes. The LeucoPatch® device, which produces a patch of fibrin platelets and leucocytes from relatively small volumes of the patient’s venous blood, overcomes many of these problems. Early pilot data suggest improved healing in a group of patients with diabetes with hard-to-heal foot ulcers.

The study population is designed to be as inclusive as possible with the aim of maximising the external validity of any findings. Thus, the intention is to include a large percentage of people with diabetes complicated by ulceration of the foot, including those with all but the most severe peripheral arterial or renal disease. Whilst patients cannot be included if the ulcer is clinically infected at the point of randomisation, should secondary infection be detected during follow-up the patient will not be withdrawn. The population of interest and likely to benefit the most are those who do not heal more than 50% in 4 weeks of observation during best standard care (Table 1), the ‘hard-to-heal’ group. It is for this reason that an initial 4-week observation period is scheduled. Whilst it is not possible to blind either the participant or their usual-care team to the allocated arm of the study, the primary outcome measure (clinical healing) will be confirmed by an observer blind to allocated treatment to reduce the possibility of bias.

The primary outcome measure is healing within 20 weeks of randomisation and the trial also includes a number of secondary outcome measures. Principal among these are rate of change in ulcer area as a predictor of the likelihood of eventual healing, minor and major amputation of the target limb, the incidence of infection and quality of life.

### Trial status

Participant recruitment commenced in August 2013. As of 31 May 2017, 595 participants consented to join the study, and of these 269 participants were randomised. Three hundred and twenty-six participants did not proceed to randomisation. Recruitment has now closed.

## Abbreviations

ABPI: Ankle-brachial Pressure Index
ADE: Adverse device effect
AE: Adverse event
DFU: Diabetic foot ulcer
DMC: Data Monitoring Committee
EGF: Epidermal growth factor
eGFR: Estimated glomerular filtration rate
EQ-5D: EuroQol-5D questionnaire (mood and function)
HRQoL: Health-related quality of life
ICF: Informed Consent Form
IDSA: Infectious Disease Society of America
ITT: Intention-to-treat
NHS: National Health Service
PDGF: Platelet-derived growth factor
PDWHF: Platelet-derived wound-healing formula
RCT: Randomised controlled trial
REC: Research Ethics Committee
SAE: Serious adverse event
SF-12: Short Form 12 questionnaire (quality of life)
TGF-β: Transforming growth factor-β
VAS: Visual Analogue Scale
VEGF: Vascular endothelial growth factor
